# Comparison of different approaches to assess fascicle length using 3D ultrasound with cadaveric dissection as a reference for the human biceps femoris long head

**DOI:** 10.1007/s40477-026-01203-2

**Published:** 2026-08-05

**Authors:** Carmela Julia Mantecón-Tagarro, Guido Weide, Yasuo Kawakami, Huub Maas

**Affiliations:** 1 Faculty of Sport Sciences, Waseda University, Saitama, Japan; 2 Human Performance Laboratory, Comprehensive Research Organization, Waseda University, Tokyo, Japan; 3 Department of Human Movement Sciences, Faculty of Behavioural and Movement Sciences and, Amsterdam Movement Sciences, Vrije Universiteit Amsterdam, Van Der Boechorststraat 7, 1081 BT Amsterdam, The Netherlands

**Keywords:** Biceps femoris, Cadaver, Hamstrings, Fascicles, Three-dimensional ultrasonography

## Abstract

**Purpose:**

The biceps femoris long head (BFlh) has a complex architecture, posing challenges for accurately assessing its fascicle length. This study compared four 3D ultrasound (3DUS)-based methods for estimating mid-belly BFlh fascicle length in human cadavers (n = 6 legs).

**Methods:**

Method 1 and Method 2 assumed straight fascicles within a single longitudinal plane, aligned with the muscle axis and oriented perpendicular to either proximal or distal aponeurosis. Method 3 represented multiplanar trajectory using two connected straight segments, whereas Method 4 further incorporated local in-plane curvature within each segment.

**Results:**

Agreement relative to the direct measurement of fascicle length by dissection using Bland–Altman bias and 95% limits of agreement (LoA), together with mean difference. All 3DUS-based estimates showed good agreement with dissection (bias < 7.5%, LoA < 18.8 mm, mean differences < 7 mm). Method 3 and Method 4 yielded the lowest bias, narrowest limits of agreement, and smallest mean differences. While repeated-measures ANOVA revealed a significant main effect, post hoc analysis did not identify significant differences between methods (all p ≥ 0.096). All methods demonstrated good intra-rater repeatability (ICC (3,1) = 0.77–0.87) and moderate measurement precision (SEM = 5.51–8.74%).

**Conclusion:**

Overall, these findings suggest that a two-plane approach yields higher accuracy for BFlh fascicle length measurements than approaches assuming straight fascicles within a single plane.

## Introduction

The long head of the biceps femoris (BFlh) contributes to knee flexion and hip extension and is frequently affected in strain injuries [1–4]. The BFlh displays a heterogeneous and often multiplanar fascicular architecture, with fascicles that are not arranged in a single plane and the proximal aponeurosis showing regional variations in shape [5–8]. These structural features can influence its mechanical function, such as maximal contraction velocity and the operating length range of the muscle [9–12].

To study BFlh muscle architecture, particularly fascicle length, several techniques have been used, including cadaveric dissection, two-dimensional ultrasound (2DUS), and more recently three-dimensional ultrasound (3DUS) [5, 6, 13]. Among these, cadaveric dissection provides direct measurements of fascicle length and remains a valuable reference [14, 15]. 2DUS is widely used to estimate fascicle length in vivo, but it assumes that fascicles run within a single plane [16, 17], which may result in erroneous estimates when fascicles run out of plane [18–20]. 3DUS addresses part of this limitation by enabling volumetric reconstructions of muscle morphology and sampling of fascicles from multiple 2D planes within the reconstructed volume. However, most studies still apply a single-plane approach rather than reconstructing the 3D fascicle trajectory [21–24].

In the BFlh, *in-vivo* freehand 3DUS has incorporated in-plane curvature within a single longitudinal view (e.g., spline-based fitting that accounts for in-plane curvature) [24]. However, single-plane approaches may remain insufficient given reported proximal aponeurosis orientation changes along the proximal–distal axis, suggesting non-planar fascicle trajectories [7, 25]. However, 3DUS-based, multi-planar approaches to assess fascicle length have not been described.

The present study aimed to compare different 3DUS-based approaches to assess BFlh fascicle length in the mid-belly region and to assess their intra-rater repeatability. Methods assuming straight fascicles within a single plane were compared with methods using a two-plane approximation of fascicle trajectory, with one method incorporating fascicle curvature. Cadaver dissection measurements served as the reference. We hypothesized that the two-plane approaches yield estimates closer to dissection rather than single-plane methods, and that accounting for intraplanar curvature would further improve agreement.

## Methods

This cadaveric study was approved by the Vrije Universiteit Amsterdam (VU) Ethics Committee (METC number: 2023.0711). Six lower limbs from three donors with no known muscular pathologies (two males and one female; age: 79 ± 3 years) were included, based on specimen availability. Specimens were obtained from the body donation program of the Department of Anatomy and Neurosciences at VU University Medical Center (VUmc), Amsterdam, The Netherlands and embalmed using the Fix-for-Life method, which preserves life-like morphology for anatomical research [26]. Donors provided written informed consent during their lifetime to participate in the body donation program. All procedures complied with local and international ethical guidelines and laws for the use of human cadaveric donors in anatomical research. Data was accessed for research purposes between 10/06/2024 and 20/07/2024.

### Three-dimensional ultrasound (3DUS)

#### Data collection—imaging protocol

The cadaveric legs were positioned in prone on a custom-made support to stabilize hip and knee alignment and to maintain the posterior thigh horizontal relative to the probe trajectory. Before US scanning, thigh length (greater trochanter to lateral femoral epicondyle), lower leg length (ischial tuberosity to fibular head), and thigh circumference (midpoint of thigh length), were recorded.

**Fig. 1:**
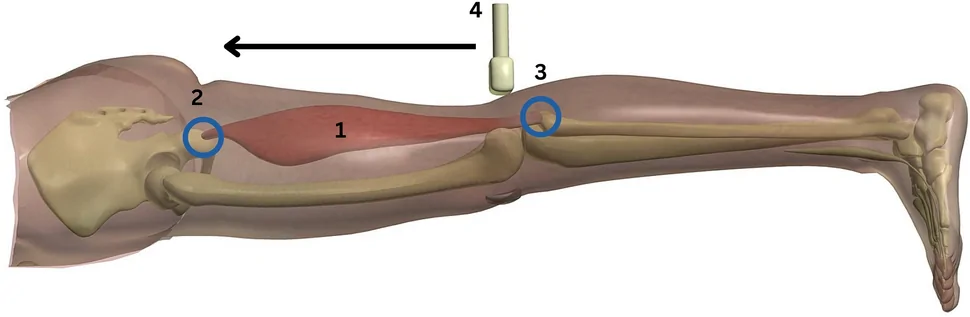
Three-dimensional ultrasound scan of the long head of the biceps femoris muscle. The scan was performed in a distal-to-proximal direction (indicated by black arrow) of the muscle (1) using an ultrasound transducer (4), with 3–4 consecutive sweeps. Each sweep started from the lateral side of the thigh and progressed toward the inner side, ensuring a 1 cm overlap between sweeps. Anatomical key structures such as the distal attachment on the fibular head (3) and proximal attachment on the ischial tuberosity (2) were included. The 3D model was created using Anim8or (freeware)

Ultrasound data were acquired using a standardized protocol adapted from Weide et al. 2017 [22] to capture three-dimensional architecture. Prior to data collection, anatomical landmarks, including the ischial tuberosity and knee condyles, were identified via palpation, verified using ultrasound, and marked on the skin using a surgical skin marker to ensure consistent probe placement. A linear transducer (L55, Hitachi Arietta Prologue, Tokyo, Japan; 5–10 MHz; bandwidth: 5–13 MHz) was used for imaging acquisition. Probe position and orientation were tracked with an optical motion capture system (NDI Polaris Vega ST, Northern Digital Inc., Waterloo, Canada) and synchronized with ultrasound acquisition using reflective markers attached to the probe and a trigger signal for time-alignment [22, 27]. For each leg, three to four longitudinal, parallel sweeps were performed at a constant speed, with ~ 1 cm overlap to ensure full coverage for 3D reconstruction (Fig. 1). Each complete sweep series was repeated multiple times (approximately 40–50 s per series). The sweep series used for reconstruction was selected based on image quality (clear superficial and deep aponeuroses, continuous fascicle striations, and minimal shadowing/dropout). When multiple series qualified, the first was analyzed.

### 3DUS reconstruction

Raw 2DUS data were aligned with the corresponding 3D probe trajectory data using custom-developed MATLAB software (R2024a; MathWorks, Natick, MA, USA), adapted from Weide et al. [22] to reconstruct a 3D voxel volume. Image intensities were interpolated into the volume, and gaps were filled using nearest-neighbor interpolation to obtain continuous reconstruction. A complete leg volume reconstruction required approximately 10 min per dataset.

Within the reconstructed volume, anatomical reference points were identified in MITK software (v2024.12, MITK, German Cancer Research Center, Heidelberg, Germany). These included:

- Ischial tuberosity (tendon of origin).
- The proximal and distal myotendinous junctions (MTJs—delimit the muscle-belly).
- The fibular head (tendon of insertion).

Anatomical landmarks were selected in a transverse view and confirmed across adjacent slices in longitudinal view [22].

The longitudinal axis of the BFlh was defined as the line connecting the ischial tuberosity to the fibular head through the central trajectory of the muscle belly and was used to guide plane orientation and fascicle tracing. Fascicle measurements were taken at 50% of the muscle belly length (mid-belly), defined as the midpoint between the proximal and distal MTJs along this axis, and used as a standardized reference. This location was selected to ensure consistent sampling across specimens and methods, to avoid transitional myotendinous regions, and because it is commonly used in single-plane ultrasound-based assessments of BFlh muscle architecture.

### Fascicle length estimation methods

**Fig. 2:**
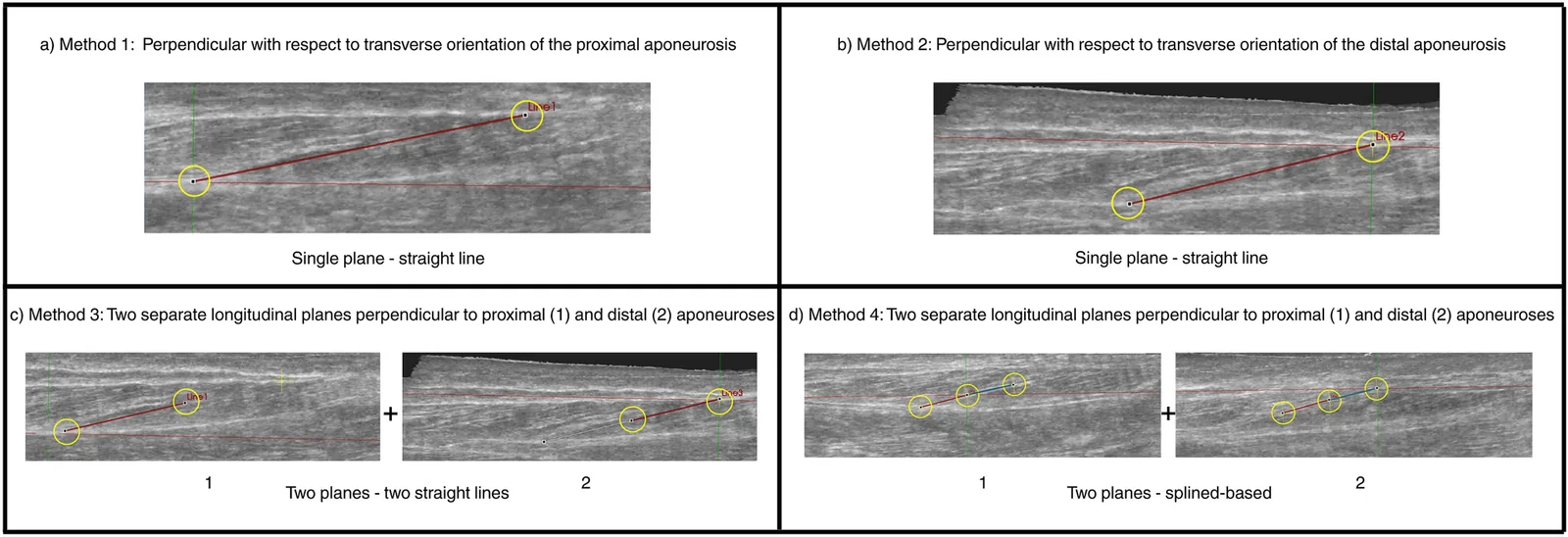
Overview of the four 3DUS-based approaches used to estimate fascicle length in the BFlh mid-belly region. **a** Method 1: Single-plane, straight-line measurement oriented perpendicular to the proximal aponeurosis (i.e. similar to 2D approach in a 3D volume). **b** Method 2: Single-plane, straight-line measurement oriented perpendicular to the distal aponeurosis (i.e. similar to 2D approach in a 3D volume). **c** Method 3: Two-plane reconstruction using two connected straight segments. **d** Method 4: Two-plane reconstruction with in-plane curvature (spline-based). Yellow circles indicate initial and endpoints of the fascicles on each approach; red lines represent the reconstructed fascicle path for each Method; blue lines represent the additional division of the fascicle on Method 4 (i.e. splined-based approach)

Within the predefined mid-belly region, one fascicle bundle portion was selected based on a priori image-quality criteria: continuous, well-defined hyperechoic striations with clearly identifiable insertions into the proximal and/or distal aponeuroses, avoiding areas with shadowing, dropout, or discontinuous patterns. The same region of interest was used across all four 3DUS-based approaches to enable within-specimen comparison. Selection was performed prior to applying method-specific measurements. All measurements were performed by the same investigator over three sessions (one week apart). If in doubt, fascicle identification and endpoint placement were checked with an independent observer. Four measurement approaches were used on the 3D volume (Fig. 2):

- **Method 1: Plane Perpendicular to Proximal Aponeurosis:**

**Fig. 3:**
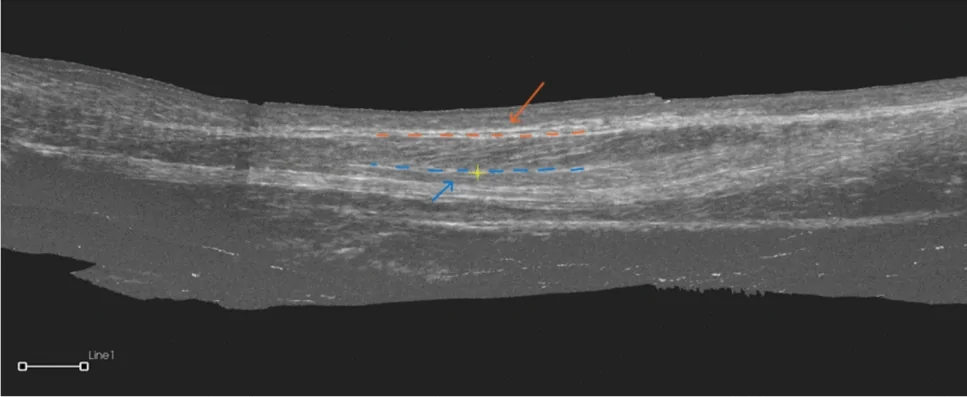
Longitudinal ultrasound image of the biceps femoris long head (BFlh) showing the mid-belly region (50% of muscle-belly length) analyzed with Method 1, in which the imaging plane was oriented perpendicular to proximal aponeurosis. The distal aponeurosis (orange dashed line) and proximal aponeurosis (blue dashed line) are indicated, and the yellow marker identifies the fascicle attachment point at the midpoint of the proximal aponeurosis. Proximal is to the left and distal to the right. Scale bar = 2 cm (20 mm)

Fascicles were assumed to run in a straight line within a single longitudinal plane (i.e. 2D approach performed on a single plane of a 3D volume), oriented relative to the muscle’s longitudinal axis and perpendicular to the local transverse plane of the *proximal* aponeurosis (Fig. 3). Within this single plane, the orientation of the hyperechoic lines representing the boundaries of the fascicles was followed from its apparent insertion on the proximal aponeurosis to its apparent termination at the distal aponeurosis. These fascicle attachment points were marked for measurement repeatability.

- **Method 2: Plane Perpendicular to Distal Aponeurosis:**

Fascicles were modeled as straight lines within a single longitudinal plane at the same mid-belly location, but oriented perpendicular to the *distal* aponeurosis. Fascicle endpoints were identified within this new plane and measured as a straight-line distance, similar to Method 1. Additionally, the midpoint of the fascicle (defined as half of its total length) was marked as a reference for Method 3.

- **Method 3: Two-plane, two-segment reconstruction:**

To account for potential non-planar fascicle trajectories, fascicles were modeled as two straight-line segments andreconstructed across two separate longitudinal planes intersecting at the predefined midpoint identified in Method 2. One plane was adjusted to be approximately perpendicular to the proximal aponeurosis and the other to the distal aponeurosis, both aligned with respect to the muscle’s longitudinal axis. Proximal and distal fascicle segments were traced separately from the midpoint to their respective aponeurotic insertions, and total fascicle length was calculated as the sum of both segments.

- **Method 4: Two-plane reconstruction with curvature fitting:**

Method 4 extended Method 3 by incorporating in-plane curvature within each segment using spline fitting [24]. Within each longitudinal plane, three control points were placed along the visible fascicle path, from the aponeurotic insertion to the midpoint, representing the fascicle’s origin, midpoint, and distal endpoint within that plane. These points allow for a curved representation within each plane. Total fascicle length was calculated as the sum of the curved segment lengths from both planes.

### Comparison between 3DUS and dissection

To enable a direct comparison between the methods, fascicle insertion points along the distal aponeurosis were matched. During cadaveric dissection, the distance from the distal end of the muscle belly to each fascicle insertion site on the distal aponeurosis was measured and expressed as a percentage of the total distal aponeurosis length. This normalized location was used to identify the corresponding fascicles in the 3DUS reconstructions. In the 3DUS volume, the distal end of the muscle belly was identified visually.

## Direct morphological measurements obtained through cadaveric dissection

**Fig. 4:**
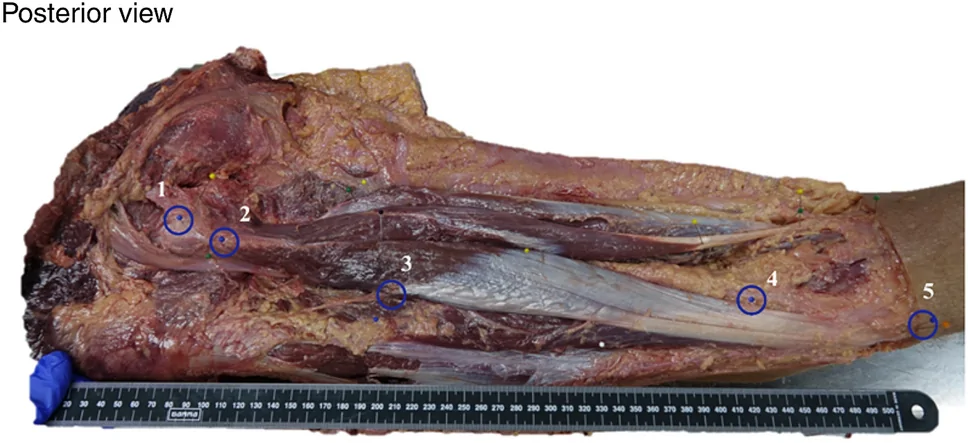
Dissected leg posterior view of the left thigh showing the long head of the biceps femoris (BFlh) with markers on relevant anatomical structures: 1) Ischial tuberosity, 2) Proximal MTJs, 3) Most proximal visible point of the distal aponeurosis, 4) Distal MTJs, and 5) Fibula head. A ruler was used for calibration

The posterior thigh was positioned in prone position and skin, adipose tissue, fascia, and gluteal muscles were removed to expose the hamstrings. Anatomical markers were identified visually and by palpation, and marker pins were placed at (Fig. 4):

- 1. Ischial tuberosity.
- 2. Proximal MTJ.
- 3. Most proximal visible point of the distal aponeurosis.
- 4. Distal MTJ.
- 5. Fibular head.

Furthermore, the following distance measurements were performed:

- a. Proximal free tendon length: from markers 1 to 2.
- b. Muscle belly length: from markers 2 to 4.
- c. Distal aponeurosis length: from markers 3 to 4.
- d. Distal free tendon length: from markers 4 to 5.
- e. Muscle–tendon unit length: from markers 1 to 5.

**Fig. 5:**
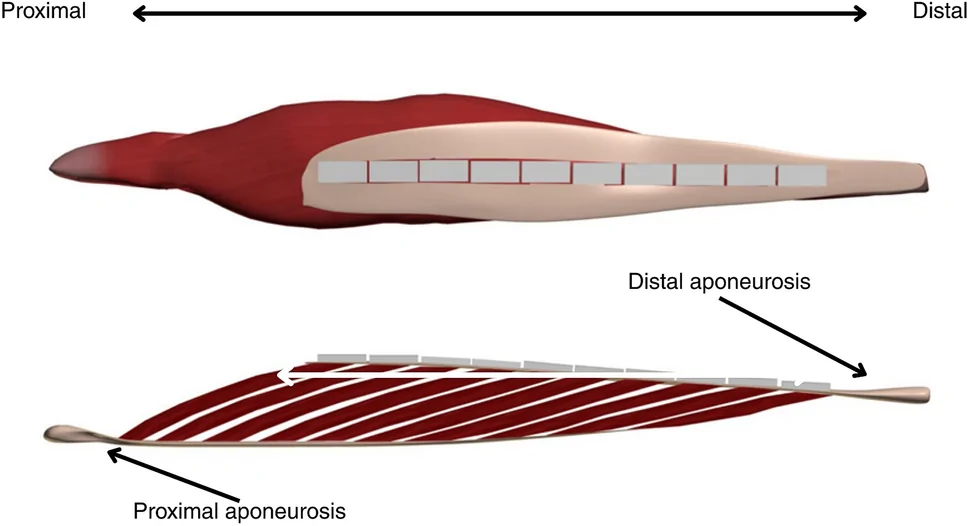
Representation of dissection process. The top image shows the posterior view of the distal (right side) and proximal (left side) ends of the distal aponeurosis of BFlh (top grey surface). On the top image, location where each fascicle section (white rectangle) was taken from the distal aponeurosis at 10% increments from 0 (left end) to 100% (right end) of its length. Bottom image, lateral view of BFlh. The fascicle’s (red diagonal line) attachment from the distal aponeurosis (top white) to the proximal aponeurosis (bottom white). The 3D model was created using Anim8or (freeware)

The BFlh was then removed for fascicle dissection. Fascicles were sampled along the midline of the distal aponeurosis at approximately 10% intervals (Fig. 5). At each location, a muscle section (approximately 1–2 cm wide and 2–3 cm long, depending on the aponeurosis length) was cut using a scalpel. Within each section, fascicles were carefully dissected from the distal to the proximal aponeurosis along their visible trajectory. Fascicle length was measured using calipers.

### Statistical analysis

Statistical analyses were performed using Jamovi (The jamovi project (2025); Version 2.6.26) and MATLAB (R2024a). Descriptive statistics (mean and standard deviation, SD) were calculated for all variables. For repeated measurements, within-method variability was quantified using the coefficient of variation (CV), expressed as a percentage.

Differences in fascicle length between the four 3DUS-based methods were assessed using repeated-measures ANOVA. When significant main effects were observed, Tukey-adjusted post hoc comparisons were performed. Effect sizes were reported as partial eta squared (η^2^ₚ).

Agreement between the 3DUS methods and dissection was assessed using Bland–Altman analysis, reporting the mean bias and 95% limits of agreement (bias ± 1.96 * SD of the differences). Proportional bias was assessed by linear regression of the differences between each 3DUS method and dissection against their mean values.

Absolute error relative to dissection was calculated for each specimen as:

$$\mid F{L}_{3DUS}-F{L}_{dissection}\mid$$

where FL_3DUS_ was fascicle length from 3DUS methods and FL_dissection_ was fascicle length from dissection (both summarized as mean ± SD).

To observe method-consistency, intra-rater repeatability across three sessions was assessed using intraclass correlation coefficients (1, 3), two-way mixed-effects model, absolute agreement). ICC (3,k) was calculated to illustrate the effect of averaging repeated measurements. ICCs were interpreted as: < 0.5 poor, 0.5–0.75 moderate, 0.75–0.9 good, and > 0.9 excellent [28].

The standard error of measurement (SEM) was calculated as:

$$SEM={SD}_{all} \times \surd (1-ICC[\mathrm{3,1}])$$

where SD_all represents the standard deviation across specimens and sessions for each method. SEM was reported in absolute and relative terms (SEM%), representing measurement precision.

The minimal detectable change at the 95% confidence level (MDC95) was calculated as:

$${MDC}_{95}=1.96 \times SEM \times \surd 2$$

and also presented in relative form (MDC%).

## Results

### Comparison of 3DUS methods

**Fig. 6:**
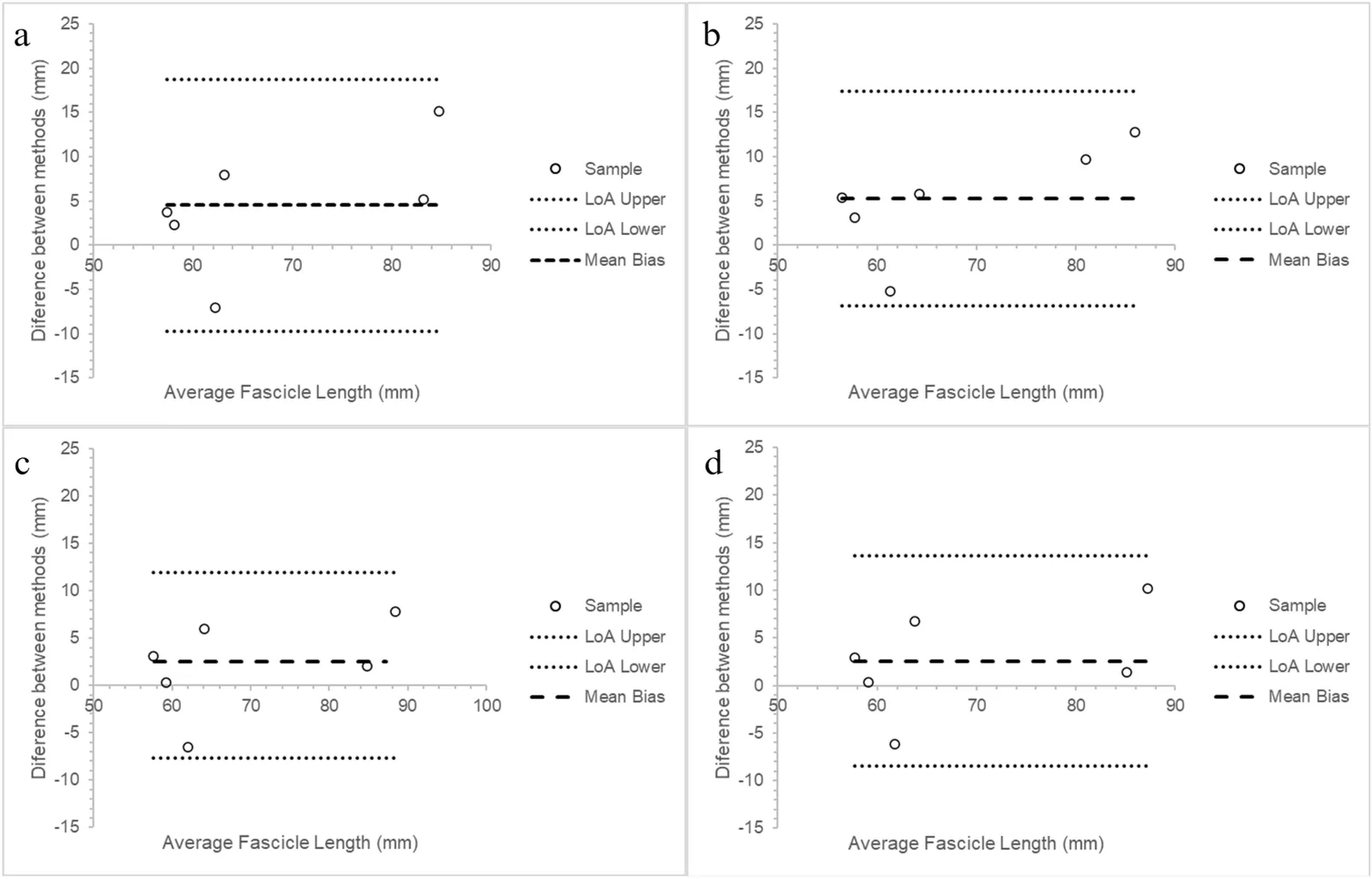
The Bland–Altman plots show the agreement between each 3DUS method and dissection, with the bias (Bias, black dashed center line) and the upper (upper limit, upper dashed line) and lower (lower limit, lower dashed line) agreement limits indicated. Each point represents a subject. Figure 6.a shows the comparison between Method 1 and Dissection. Figure 6.b shows the comparison between Method 2 and Dissection. Figure 6.c shows the comparison between Method 3 and Dissection. Figure 6.d shows the comparison between Method 4 and Dissection

Fascicle length estimates for the four 3DUS-based approaches are summarized in Table 1 (*n* = 6 legs). Agreement with dissection is reported in Table 2 and Fig. 6. Mean bias ranged from 2.1 mm (3.0%; Method 4) to 5.3 mm (7.5%; Method 2), expressed relative to the mean dissection fascicle length across specimens (70.4 mm). The two-plane methods (Method 3 and 4) showed the lowest bias, narrowest limits of agreement, and smallest differences compared with dissection. Incorporating fascicle curvature (Methods 4) resulted in slightly improved agreement compared with straight two-plane approach (Method 3). Mean differences between 3DUS and dissection ranged from 4.3 ± 2.9 mm (Method 4) to 7.0 ± 3.6 mm (Method 2). No significant proportional bias was detected for any method (all *p* > 0.05). Repeated-measures ANOVA showed a significant main effect of the method (F (3, 15) = 5.48, *p* = 0.010, η^2^ₚ = 0.52). However, post hoc comparisons revealed no significant pairwise differences between 3DUS-based methods (all *p* ≥ 0.096; Table 3).

**Table 1:** Fascicle location and fascicle length measured by dissection and four 3DUS-based methods

	Fascicle location on distal aponeurosis (%)	FL dissection (mm)	FL Method 1 (mm)	FL Method 2 (mm)	FL Method 3 (mm)	FL Method 4 (mm)
S1_RL	57.9 ± 4.3	59.2 ± 0.9	55.5 ± 2.1	53.8 ± 2.7	56.3 ± 3.8	56.1 ± 3.8
S1_LL	63.4 ± 5.2	59.3 ± 1.4	57.0 ± 0.8	56.2 ± 1.3	59.0 ± 2.3	59.0 ± 1.8
S2_RL	50.7 ± 1.2	58.7 ± 2.0	65.8 ± 2.1	63.9 ± 7.0	64.9 ± 6.5	65.2 ± 5.3
S2_LL	45.2 ± 6.7	67.1 ± 1.4	59.2 ± 8.5	61.3 ± 8.96	60.4 ± 7.5	61.1 ± 6.4
S3_RL	56.8 ± 4.5	85.8 ± 2.0	80.6 ± 3.9	76.1 ± 1.4	84.4 ± 9.1	83.7 ± 6.8
S3_LL	61.8 ± 3.9	92.3 ± 1.0	77.2 ± 1.1	79.5 ± 4.0	82.1 ± 6.3	84.5 ± 6.0
Mean (all legs)	56.0 ± 6.6	70.4 ± 15.5	65.9 ± 10.3	65.1 ± 10.5	67.8 ± 12.3	68.3 ± 12.7

Mean fascicle location (as a percentage of distal aponeurosis length) and fascicle length (FL, mm) obtained from dissection (reference) and four 3D ultrasound–based approaches (Method 1 = plane perpendicular to proximal aponeurosis; Method 2 = plane perpendicular to distal aponeurosis; Method 3 = two-plane reconstruction; Method 4 = two-plane reconstruction with fascicle curvature), for both lower limbs (right [RL] and left [LL]) of three donors (S1–S3). Values are reported as mean ± standard deviation (SD) across repeated measurements (*n* = 3). The final row (Mean across legs) represents the mean ± SD across all six legs (between-specimen variability). Differences in FL estimates across 3DUS approaches were examined using a repeated-measures ANOVA (p > 0.01; η^2^ₚ = 0.52)

*FL* fascicle length; *S1* sample 1; *S2* sample 2; *S3* sample three; *RL* right limb; *LL* left limb; *SD* standard deviation

**Table 2:** Agreement analysis: differences between 3DUS approaches and dissection

	Bias (mm)	95% upper LoA (mm)	95% lower LoA (mm)	Mean difference (mm)	Mean Difference (%)
Method 1	4.5 ± 7.3	18.8	-9.7	6.9 ± 4.5	6.4
Method 2	5.3 ± 6.2	17.4	-6.8	7.0 ± 3.6	7.5
Method 3	2.6 ± 5.6	13.6	-8.5	4.6 ± 3.7	3.7
Method 4	2.1 ± 5.0	11.9	-7.7	4.3 ± 2.9	3.0

Agreement metrics between fascicle length (FL) estimates obtained with four 3DUS-based approaches and the reference (dissection). Values are reported as mean bias ± standard deviation (SD) and 95% limits of agreement (LoA = bias ± 1.96 × SD), expressed in millimeters (mm). Mean difference values are presented in millimeters (mean ± SD). Percentage values represent the mean bias expressed relative to the mean dissection fascicle length across specimens (70.4 mm)

*FL* fascicle length, *SD* standard deviation, *LoA* limits of agreement

**Table 3:** Tukey-adjusted post hoc pairwise comparisons for fascicle length between 3DUS-based methods

Comparison	Mean difference (mm)	SE	df	t	p (Tukey)
Method 1 – Method 2	0.09	0.10	5.00	0.85	0.83
Method 1 – Method 3	-0.22	0.08	5.00	− 2.70	0.14
Method 1 – Method 4	-0.26	0.12	5.00	− 2.25	0.23
Method 2 – Method 3	-0.31	0.12	5.00	− 2.46	0.18
Method 2 – Method 4	-0.35	0.11	5.00	− 3.05	0.10
Method3 – Method 4	-0.04	0.06	5.00	− 0.80	0.85

Pairwise comparisons of fascicle length (FL, mm) between four 3D ultrasound–based approaches (Method 1 = plane perpendicular to proximal aponeurosis; Method 2 = plane perpendicular to distal aponeurosis; Method 3 = two-plane reconstruction; Method 4 = two-plane reconstruction with fascicle curvature), following repeated-measures ANOVA. Mean differences (Method A − Method B), standard errors (SE), degrees of freedom (df), t-values, and Tukey-adjusted p-values are reported (n = 6 legs). No pairwise comparisons reached statistical significance (all *p* ≥ 0.096)

*FL* fascicle length, *SE* standard error, *df* degrees of freedom

### Intra-rater repeatability

All 3DUS-based methods showed good-to-excellent intra-rater repeatability metrics (Table 4). ICC (3,1) ranged from 0.77 to 0.87, while the ICC (3,k) was above 0.90 for all methods.

**Table 4:** Intra-rater repeatability metrics for each 3DUS Method

	ICC (3,1)	ICC (3,k)	SEM (mm)	SEM (%)	MDC_95_	MDC_95_ (%)	CV (%)
Method 1	0.87	0.95	3.63	5.51	10.05	15.26	15.69
Method 2	0.81	0.93	4.53	6.96	12.55	19.28	16.07
Method 3	0.77	0.91	5.92	8.74	16.41	24.19	18.16
Method 4	0.78	0.91	5.97	8.74	16.55	24.24	18.60

Intra-rater repeatability of fascicle length (FL) measurements obtained with the four three-dimensional ultrasound (3DUS) methods

Repeatability was determined using two-way mixed-effects intraclass correlation coefficients for absolute agreement (ICC(3,1) for single measurements; ICC(3,k) for the average of three sessions)

The standard error of measurement (SEM) and minimal detectable change at 95% confidence (MDC₉₅) were expressed in both absolute (mm) and relative (%) terms

MDC₉₅ was calculated as 1.96 × SEM × √2

The coefficient of variation (CV, %) was calculated as the coefficient of variation of repeated measurements (SD/mean × 100) across sessions for each method

All four methods demonstrated good to excellent intra-rater repeatability, with slightly greater variability in Method 3 and 4 due to its higher geometric complexity

*FL* fascicle length; *ICC* intraclass correlation coefficient; *SEM* standard error of measurement; *MDC₉₅* minimal detectable change at 95% confidence; *CV* coefficient of variation

SEM ranged from 3.6 mm to 6.0 mm (5.6–8.7%). The MDC95 ranged from 10.0 mm to 16.6 mm (15.3–24.2%). CV ranged from 15.7% to 18.6%, respectively.

## Discussion

This study compared four different 3DUS-based approaches to estimate BFlh fascicle length in human cadavers. Consistent with our hypotheses: (a) approaches accounting for multiplanar fascicle trajectories showed more accurate fascicle length estimates than single-plane approaches; and (b) incorporating fascicle curvature showed a limited additional improvement. All methods demonstrated good to excellent intra-rater repeatability.

### Comparison of different approaches to assess fascicle length using 3DUS

Agreement between 3DUS-based and dissected fascicle lengths on this study was comparable to previous reports in other muscles. Benard et al. (2009) and Barber et al. (2009) reported mean differences between 3DUS and dissection for medial gastrocnemius of 1–3 mm (3–4%) while Haberfehlner et al. (2016) reported 1.0 ± 0.9 cm (8.9%) for semitendinosus. Overall, 3DUS can provide fascicle length estimates close to dissection; however, agreement varies across muscles and methods, underscoring the need for muscle-specific method selection and validation.

In the present study, two-plane approaches showed better agreement with dissection than single-plane methods, with smaller bias and differences, and narrower limits of agreement. Proportional bias was not detected, indicating that differences relative to dissection did not depend on fascicle length. When comparing the four 3DUS-based methods directly, repeated-measures ANOVA revealed a significant main effect; however, post hoc tests did not identify significant differences. These results suggest that while mean fascicle length estimates were similar across 3DUS methods, the choice of measurement approach (single-plane vs two-plane) influences accuracy relative to the dissected reference. Because fascicles may not lie entirely within a single imaging plane, sampling across two planes may better capture their trajectory and may explain the slightly shorter estimates observed with single-plane methods (i.e. as 2D analysis).

Accounting for fascicle curvature (Method 4) provided a limited improvement compared with the straight two-plane approach (Method 3). Non-linear curve fascicle paths, which have been described previously, can refine length estimates when the visible fascicle path deviates from a straight line [12, 20]. However, in the present dataset, the difference between Methods 3 and 4 was small (≈0.3 mm) and likely within typical variability of manual point placement, suggesting limited added value under the present conditions.

### Intra-rater repeatability of 3DUS-based measurements

All four 3DUS-based methods showed good to excellent intra-rater repeatability, consistent with previous studies in other muscles. For example, Weide et al. (2017) and Haberfehlner et al. (2016) reported similar repeatability for 3DUS applied to the vastus lateralis (ICC (3,3) = 0.98) and the distal fascicles of the semitendinosus (ICC (single measurement) = 0.75), noting that ICC values can vary depending on the model and whether repeated measurements are averaged.

In our present study, variability indices (SEM, MDC₉₅, CV) were slightly higher for two-plane than single-plane approaches. This likely reflects the increased methodological complexity by the greater number of manual decisions. The two-plane methods require the independent alignment of two longitudinal planes (each referenced to a different aponeurosis) and identifying a common midpoint to combine segments. Inconsistencies in plane orientation or midpoint definition may therefore affect fascicle length estimates. Additionally, Method 4 requires placement of additional control points to assess fascicle curvature, which may further increase variability.

For the BFlh, Pincheira et al. (2021) reported good repeatability for a 3DUS-based approach on a single longitudinal plane (ICC(3,k) = 0.85). Our findings extend this work by directly comparing single-plane and multiplanar approaches against dissected reference measurements, supporting that 3DUS-based fascicle length assessments can be repeatable.

### Implications on fascicle length estimation

Differences between the single-plane and two-plane approaches were relatively small in the mid-belly region. Anatomical studies have demonstrated that BFlh architecture varies along its length, with region-specific differences in fascicle arrangement [6]. Additionally, cadaveric investigations have shown structural transitions within the proximal and distal tendon–aponeurosis complex and regional variation in the morphology of the proximal aponeurosis [25, 29]. Because fascicles attach to these aponeurotic structures, this may lead to regional differences in the fascicle architecture. In regions where local anatomy is relatively uniform, a simplified single-plane representation may yield sufficiently accurate estimates. However, larger methodological differences may emerge in proximal or distal regions, in which case multi-plane approaches may be more appropriate.

### Strengths and limitations

The strength of this study is the direct comparison of multiple 3DUS-based analysis approaches against dissected fascicle length measurements in the same BFlh specimens, providing a benchmark for method selection. Repeatability was also evaluated using standardized workflow, supporting application in studies requiring within-operator consistency.

Several limitations should be noted. The sample size (six legs) was small, limiting the statistical power and generalizability. This study was not designed to evaluate between-specimen differences or regional variation across the muscle, and inter-individual anatomical variability may not be fully captured. All measurements were performed by a single tester, thus only intra-rater repeatability was assessed and inter-rater variability remains unknown. In addition, the use of Fix-for-Life embalming method [26] may have influenced tissue mechanical and acoustic properties compared with in vivo conditions and limiting comparison with other cadaveric studies. Finally, only the mid-belly region was examined; future studies should include other regions.

## Conclusions

All 3DUS based approaches to assess fascicle length in the Biceps Femoris long head showed good to excellent intra-rater repeatability. The estimates of fascicle length using a two-plane approach differed only to a limited degree to those using a single-plane approach. Differences may be greater in other, more proximal or distal, regions of the muscle, but this requires further investigation.

## Data Availability

The data that support the findings of this study are available from the corresponding author upon reasonable request.

## Abbreviations

2DUS: Two-dimensional ultrasound
3DUS: Three-dimensional ultrasound
BFlh: Long head of the biceps femoris
CV: Coefficient of variation
ICCs: Intraclass correlation coefficients
LoA: 95% Limits of agreement
MDC95: Minimal detectable change at the 95% confidence level
MDC%: Minimal change detectable beyond the measurement error of each method
MTJs: Myotendinous junctions
SD: Standard deviation
SE: Standard errors
SEM: Standard error of measurement
VUmc: VU University Medical Center
η^2^ₚ: Partial eta squared
